# Evaluating Assistive Technology Outcomes in Boccia Athletes with Disabilities Using AI-Based Kinematic Analysis

**DOI:** 10.3390/bioengineering12070684

**Published:** 2025-06-23

**Authors:** Wann-Yun Shieh, Yan-Ying Ju, Shiu-Yuan Yang, I-Chun Chen, Hsin-Yi Kathy Cheng

**Affiliations:** 1 Department of Computer Science and Information Engineering, College of Engineering, Chang Gung University, Taoyuan 333, Taiwan; wyshieh@mail.cgu.edu.tw; 2 Department of Physical Medicine and Rehabilitation, Chang Gung Memorial Hospital, Taoyuan 333, Taiwan; 3 Department of Adapted Physical Education, National Taiwan Sport University, Taoyuan 333, Taiwan; yanju@ntsu.edu.tw; 4 Department of Sport Medicine Research, Taiwan Institute of Sports Science, Kaohsiung 813, Taiwan; 5 Graduate Institute of Early Intervention, College of Medicine, Chang Gung University, Taoyuan 333, Taiwan; as91098607@gmail.com (S.-Y.Y.); burnzoe.tw@gmail.com (I.-C.C.)

**Keywords:** assistive technology, adaptive sports, AI-based motion analysis, pose estimation, boccia, inclusive sport

## Abstract

This study explores how artificial intelligence (AI) can support the evaluation of assistive technology outcomes in adaptive sports, focusing on elite boccia athletes with disabilities. Using a multi-stage motion analysis framework, we integrated OpenPose, ViTPose, and Lifting to estimate seated joint kinematics with greater precision. Match footage from 12 athletes at the 2018 Asia-Pacific Boccia Open was analyzed across five biomechanical phases: preparation, acceleration, peak, release, and follow-through. AI-enhanced 2D and 3D pose estimation methods were applied to assess throwing strategies and motor variability. ViTPose outperformed OpenPose in joint detection accuracy (F1-score: 85% vs. 79.5%), while Lifting improved 3D estimation by reducing joint position error by 16%. Principal Component Analysis revealed greater movement consistency in overhand throws compared to underhand techniques. The proposed pipeline provides an interpretable and scalable method for measuring performance, motor control, and strategy-specific movement outcomes in boccia, offering practical applications for evidence-based coaching, athlete classification, and the design of inclusive assistive sport technologies.

## 1. Introduction

Boccia is a target-oriented sport that demands a high level of throwing accuracy and control, designed for individuals with severe physical disabilities, particularly those with cerebral palsy or muscular dystrophy. As an official Paralympic event, boccia not only provides opportunities for athletic participation but also supports improvements in motor control, cognitive function, and psychological well-being [1,2]. Boccia can be played in individual, pair, or team formats. The objective of the game is to throw leather balls—colored red or blue—as close as possible to a white target ball (the jack). The game is played on a 12.5 m × 6 m court, with an additional 2 m of in-bounds playable space around it. Due to its reliance on precision and movement control, accurate biomechanical evaluation of throwing performance is essential for performance optimization [3,4,5]. However, there remains a lack of validated and accessible technologies for objectively assessing movement in this population, posing a significant barrier to evidence-based coaching and inclusive performance assessment.

In recent years, adaptive sports have gained increasing attention from international policy and sports organizations [6,7,8]. Numerous governmental and non-governmental entities have introduced policies to promote accessibility and inclusion for athletes with disabilities. Nonetheless, unlike mainstream sports, boccia athletes have limited access to motion capture systems and biomechanical analysis tools, which limits their ability to benefit from assistive technology innovations in sports performance measurement. This gap in accessibility and measurement infrastructure directly affects training precision, injury prevention, and classification equity. It highlights the urgent need to apply modern computational technologies to assess movement patterns and improve training methodologies.

Pose estimation technologies have made substantial progress in recent years and have been successfully applied in sports such as basketball and track and field [9,10,11,12]. However, research focused on athletes with disabilities—particularly those involved in seated and wheelchair-based sports—remains limited [13]. Most existing studies concentrate on able-bodied athletes, overlooking the unique movement characteristics of boccia players, whose mobility is often constrained. The absence of measurement tools tailored to seated biomechanics underscores a critical barrier to ensuring equitable outcomes in adaptive sport performance assessment.

Currently, common pose estimation tools include OpenPose, ViTPose, and Lifting. OpenPose [14] estimates body posture using 2D keypoint detection and is widely applied in sports science. However, it was originally developed for standing movements and may suffer from reduced accuracy when applied to seated athletes, due to changes in camera angles and limited limb mobility [15,16]. In addition, many adaptive athletes exhibit highly refined upper-limb control and require strong core stability, necessitating more advanced tools for precise motion analysis. We selected OpenPose for its proven robustness in real-world sports applications and its ability to provide reliable initial keypoint estimates in complex environments, such as wheelchair sports competitions.

ViTPose, a recently developed pose estimation method based on Vision Transformer architecture, offers higher keypoint detection accuracy than OpenPose [17]. Utilizing a self-attention mechanism [15], ViTPose effectively captures long-range dependencies between joints, making it especially robust under occlusion, complex backgrounds, and changing viewpoints. We chose ViTPose because seated throwing in boccia often involves occluded joints (such as the shoulder and wrist during acceleration phases), and the ViTPose architecture is particularly suited for handling such challenges. In seated sports analysis, ViTPose enables more precise recognition of upper-limb movement patterns and reduces angle distortion errors.

Lifting addresses this challenge by converting 2D keypoints into 3D joint coordinates, overcoming the limitations of purely 2D systems [18]. It is particularly suitable for non-standard movements, such as seated throwing, as it reduces the impact of perspective distortion and occlusion. In boccia applications, Lifting could enhance the objective measurement of critical motion features such as shoulder rotation, hand trajectory, and trunk stability—thereby supporting more inclusive and individualized training interventions. We selected Lifting because it allows for robust 3D reconstruction from 2D data without the need for specialized hardware, making it an accessible and scalable solution for biomechanical analysis in adaptive sports contexts.

The combined use of OpenPose, ViTPose, and Lifting was thus intentionally designed to address the specific challenges of analyzing wheelchair athletes in boccia: seated posture, occlusions, atypical limb movements, and the need for a low-cost, competition-compatible solution.

This study aims to bridge the current research gap by developing an AI-based pose estimation method tailored to the biomechanics of boccia athletes. By incorporating machine learning techniques, the method seeks to enhance the accuracy of motion analysis and potentially optimize athlete training. The integration of OpenPose, Lifting, and ViTPose is expected to improve the precision of seated motion analysis and offer more detailed, personalized biomechanical insights for athletes with disabilities—thus providing a data-driven foundation for evaluating assistive sports technologies and supporting equitable performance assessment in adaptive sports.

## 2. Materials and Methods

**Figure 1:**
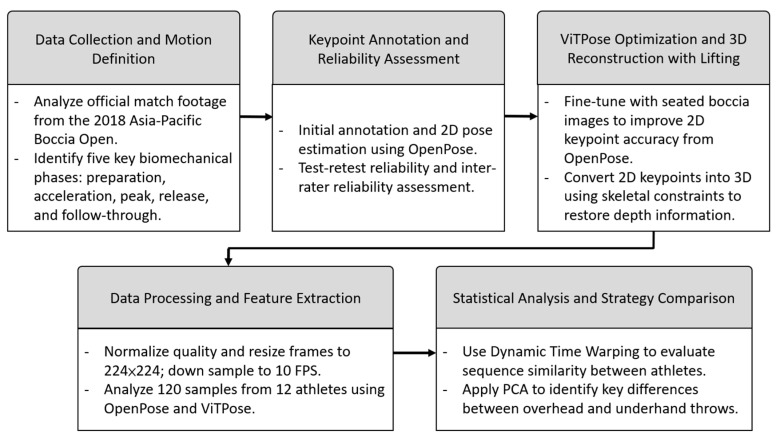
A schematic overview of the five-stage analytical framework.

This study followed a five-stage analytical framework (Figure 1), covering data collection and motion definition, AI-based pose estimation and optimization, spatial reconstruction, feature extraction, and strategy comparison.

### 2.1. Stage 1: Data Collection and Motion Definition

**Figure 2:**
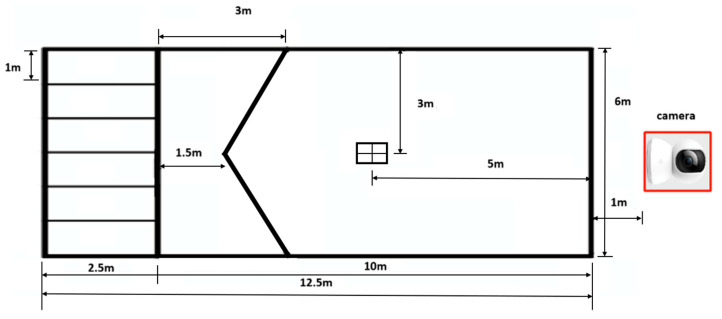
Layout of the competition court and camera placement used for motion capture.

This study analyzed official match footage from the 2018 Asia-Pacific Boccia Open, including a total of 12 elite athletes from BC1 and BC2 classifications. BC1 and BC2 are international boccia classification categories defined by the severity and type of physical disability. BC1 athletes typically have more severe motor impairments and may use assistive devices or require a sport assistant, whereas BC2 athletes have greater functional ability and do not use assistance during play. Among the 12 athletes, 6 used overhand throwing strategies and the other 6 used underhand techniques, with all performing right-handed throws from a seated position. The footage was recorded using fixed-position full HD cameras (1080p resolution) (Sony DSC-RX100M2, Sony Corporation, Tokyo, Japan) at a frame rate of 60 frames per second (FPS), as provided by the official tournament broadcast. The competition venue and camera placement are illustrated in Figure 2. Drawing on analytical methods from other throwing sports (e.g., baseball) [19], each athlete’s motion was divided into five key biomechanical phases: Arm Cocking Phase, Start of Acceleration Phase, Apex of Swing, Release and Initial Follow-Through, and Final Position. One representative frame was extracted from each phase, resulting in five images per athlete for analysis.

### 2.2. Stage 2: Keypoint Annotation and Reliability Assessment

Initial 2D pose estimation was performed using OpenPose (Carnegie Mellon University, Pittsburgh, PA, USA). The five key posture frames per athlete, identified in the previous stage, were manually annotated by two researchers experienced in wheelchair throwing using Paint X Lite to calibrate the OpenPose outputs. If automatic estimations significantly deviated from actual postures, adjustments were made collaboratively; in case of disagreement, a third expert adjudicated. Annotation reliability was assessed using intraclass correlation coefficients (ICC), yielding an intra-rater reliability of 0.89 and an inter-rater reliability of 0.83.

### 2.3. Stage 3: ViTPose Optimization and 3D Reconstruction with Lifting

**Figure 3:**
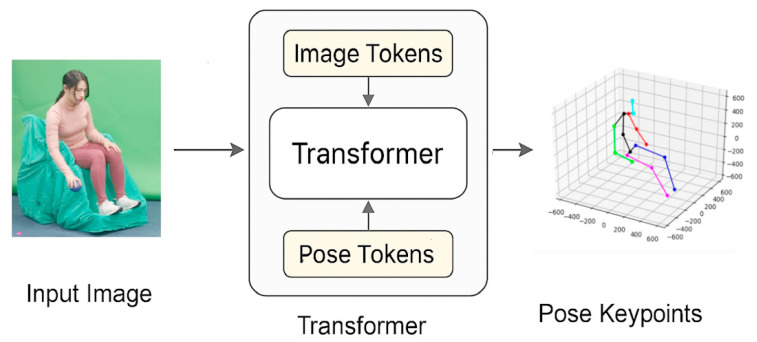
Sample training images for ViTPose fine-tuning under seated boccia conditions.

ViTPose (Institute of Automation, Chinese Academy of Sciences, Beijing, China), based on Vision Transformer architecture, was adopted to enhance keypoint detection accuracy under seated conditions and occlusion. The ViTPose model was initialized using OpenPose outputs and fine-tuned on a supplementary dataset of 60 images depicting seated boccia throwing, which was independently collected by the research team under controlled laboratory conditions. These images featured athletes in various postures and lighting conditions to enhance recognition robustness (Figure 3). The optimized 2D keypoints were then passed into the Lifting model for 3D spatial reconstruction. Lifting (University of British Columbia, Vancouver, BC, Canada) incorporated skeletal geometry and link constraints to compensate for missing depth data and enhance the spatial analysis of movements. The mathematical representation of keypoint detection in Lifting is as follows:

$$\left\{\left(x_{p},c_{p}\right)\right\}\begin{array}{c} p\\ p=1 \end{array}=d\left(I\right),\;I\in R^{w\times h\times 3},\;x_{p}\in R^{2},\;c_{p}\in \left[0,1\right]$$

whereas

- $I\in R^{w\times h\times 3}$: The input image, where *w* and *h* represent the width and height, respectively, and 3 denotes the RGB color channels.
- $d\left(.\right)$: The keypoint detector (e.g., OpenPose), which processes the input image *I* and predicts the keypoints.
- $x_{p}\in R^{2}$: The 2D coordinates (x,y) of the *p*-th keypoint.
- $c_{p}\in \left[0,1\right]$: The confidence score for the *p*-th keypoint, representing the reliability of the detection.
- *P*: The total number of detected keypoints (e.g., 17 for a standard human pose model).

### 2.4. Stage 4: Data Processing and Feature Extraction

All videos had a resolution of 1080p and a frame rate of 60 FPS. Actions were reviewed and annotated at 0.5× speed using QuickTime Player. Preprocessing included contrast and brightness normalization, and all images were resized to 224 × 224 pixels to match ViTPose input requirements. Footage was down sampled to 10 FPS to reduce redundancy. In total, 120 pose data samples (12 athletes × 5 frames × 2 systems) were processed for joint angle analysis.

### 2.5. Stage 5: Statistical Analysis and Strategy Comparison

Dynamic Time Warping (DTW) was employed to assess motion stability and sequence similarity across athletes. Principal Component Analysis (PCA) was used to identify key biomechanical factors contributing to variability and to compare biomechanical characteristics and individual differences between overhand and underhand throwing strategies.

This study was approved by the Institutional Review Board (IRB) of National Taiwan Normal University (IRB Number: 201712EM003). As the research involved analysis of publicly available competition footage and all subjects participated in a public sporting event, the requirement for individual informed consent was waived by the IRB.

## 3. Results

### 3.1. Identification of Key Throwing Phases

This study analyzed throwing movements from 12 elite athletes who participated in the 2018 Asia-Pacific Boccia Open. Participants were classified into BC1 and BC2 categories and grouped by throwing strategy (overhand vs. underhand) to ensure diversity and representativeness of disability profiles and assistive needs. Six athletes used overhand throws and six used underhand throws; all were right-handed and performed seated throws.

**Figure 4:**
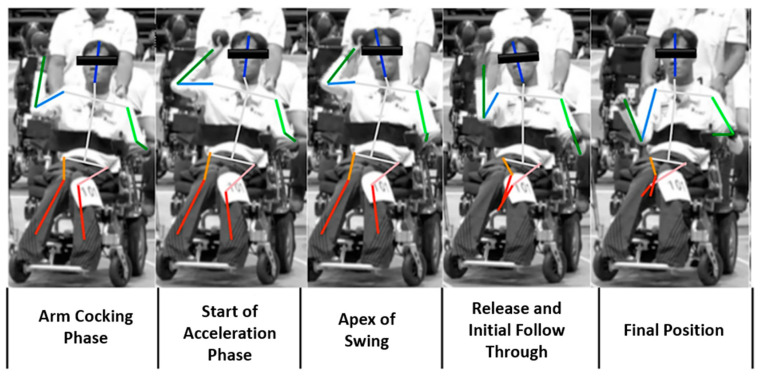
Key movement features identified in seated boccia throwing. Arm cocking phase (preparation): the athlete holds the ball while focusing on the target. The upper limb is slightly flexed, marking the beginning of the swing motion. Start of acceleration phase: the arm reaches maximum backward extension; shoulder and elbow angles show clear changes, initiating acceleration. Apex of swing: the motion transitions to forward movement. The shoulder and elbow reach maximal extension in preparation for release. Release and initial follow-through: the ball is released from the hand, accompanied by a natural forward swing and compensatory lower limb stabilization, initiating the follow-through. Final position: the throwing arm returns to a resting or stabilized position, indicating completion of the motion.

Based on established principles in sports biomechanics and seated adaptive movement analysis, each motion was segmented into five key biomechanical phases, with one representative frame extracted per phase for each athlete. These phases, defined according to biomechanical and kinematic criteria, are illustrated in Figure 4 and described below:

These five phases formed the analytical basis for standardized pose estimation using OpenPose and Lifting and served as a reference for performance assessment and individualized coaching.

### 3.2. Statistical Analysis of Throwing Patterns

To evaluate movement consistency and inter-individual variability, Principal Component Analysis (PCA) was applied to the joint angle data extracted from the key phases. The results demonstrated clear clustering across athletes, indicating distinct movement strategies.

**Figure 5:**
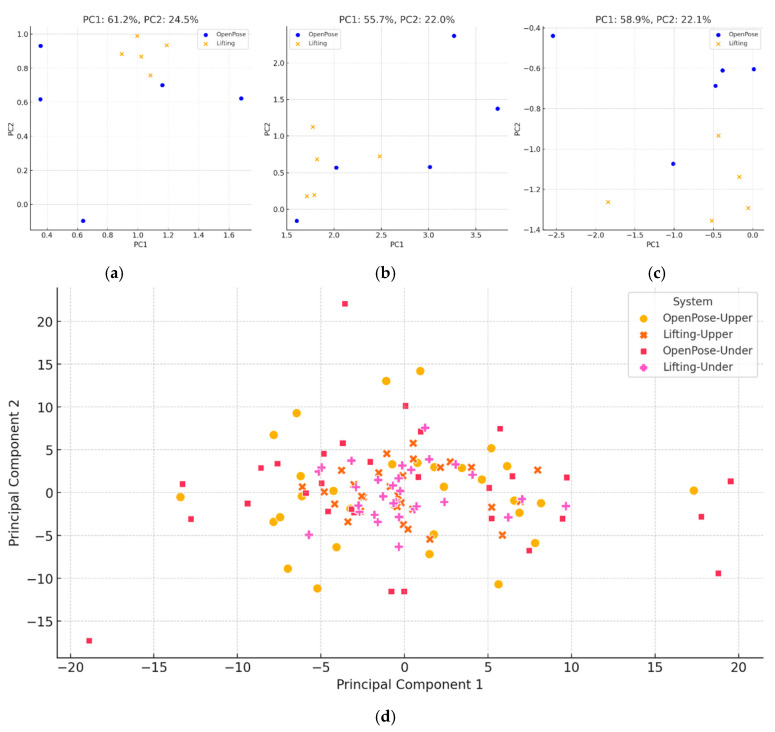
Principal component analysis of joint angle variability in seated boccia throws: (**a**) athlete A; (**b**) athlete C; (**c**) athlete D; (**d**) all athletes.

For instance, Athlete A (overhand) showed highly concentrated data points in the PC1–PC2 plane, where PC1 explained 61.2% and PC2 explained 24.5% of the total variance (Figure 5a). This suggests the majority of the variation stemmed from elbow extension and shoulder abduction. In contrast, Athlete C (overhand) exhibited more scattered data, with PC1 explaining 55.7%, reflecting less consistent control over wrist and shoulder movement (Figure 5b).

Underhand athletes showed greater variability across subjects. For instance, Athlete D’s PCA results showed PC1 accounting for 58.9% (shoulder rotation), and PC2 for 22.1% (wrist deviation) (Figure 5c). Overall, underhand throwing strategies showed more scattered PCA projections, indicating less consistent motor control than overhand strategies.

Comparisons between OpenPose and Lifting outputs showed that Lifting consistently yielded tighter PCA clusters, suggesting higher angular measurement stability. For example, Athlete A’s shoulder abduction standard deviation was 6.8° using OpenPose and 3.5° using Lifting.

Figure 5d visualizes PCA clusters across all athletes, where cross and plus markers denote Lifting-derived data, which were more clustered, especially in overhand strategies. This suggests Lifting provided more stable pose estimates, particularly under seated and occluded conditions.

### 3.3. Application of Lifting and OpenPose in Seated Throwing Analysis

To quantify system performance, standard deviations of three joint angles (shoulder, elbow, and wrist) were calculated across the five biomechanical phases (Table 1). Among overhand athletes, Lifting showed significantly lower variability: shoulder (3.5° vs. 6.8° with OpenPose), and elbow (3.1° vs. 6.5°).

In overhand athletes, Lifting reduced elbow joint variability by 52%, highlighting its ability to provide stable angular tracking. A similar pattern was observed in underhand athletes: shoulder (3.9° vs. 7.2°), elbow (3.5° vs. 7.4°), and wrist (3.2° vs. 6.8°), confirming Lifting’s robustness across throwing styles.

Across all conditions, Lifting consistently reduced angular variability, supporting its application for delivering individualized biomechanical feedback within adaptive sports contexts. This has implications for coaching precision and athlete monitoring in seated competitions.

While OpenPose occasionally misestimated joint positions—especially during the release and follow-through phases due to occlusion and seated posture constraints—Lifting applied anatomical constraints and skeletal priors to compensate for missing or misaligned keypoints. This enabled more accurate reconstruction of motion dynamics, which is essential for applications in adaptive performance tracking. These findings validate the utility of Lifting as an assistive technology tool for analyzing seated throwing patterns, supporting evidence-based athlete development, classification, and inclusive training design.

## 4. Discussion

This study demonstrated the feasibility and practical value of applying AI-driven motion analysis in adaptive sports by developing an integrated pose estimation pipeline tailored to seated boccia athletes. By combining OpenPose, ViTPose, and Lifting, the system achieved high-precision kinematic reconstruction under conditions of occlusion and reduced mobility—common challenges in wheelchair-based sports. Compared to traditional pose estimation tools, the multi-stage workflow yielded improvements in angular stability and joint tracking accuracy, particularly in shoulder and elbow movements.

A key contribution of this study was the segmentation of boccia throwing into five biomechanical phases, creating a scalable framework for performance analysis. Among the three estimation systems, the ViTPose–Lifting combination consistently outperformed OpenPose in both keypoint detection and 3D reconstruction. This advantage was most apparent during high-velocity or occluded movements such as elbow extension, highlighting the suitability of transformer-based models for adaptive biomechanics.

Principal Component Analysis (PCA) revealed that overhand throwers exhibited tighter clustering of joint angle data than underhand throwers, particularly along dominant joint axes like shoulder abduction and elbow extension. While this finding might suggest overhand throwing to be a “more stable” technique, it is crucial to interpret this in the context of functional capacity, not performance preference. In boccia, an athlete’s choice of throwing strategy is largely dictated by their physical condition, such as trunk control, range of motion, or muscle tone. Therefore, the benefit of PCA lies not in ranking throwing styles but in identifying the joints that contribute to movement variability within each strategy. Coaches and rehabilitation specialists can use this information to tailor interventions that improve control in variable joints, regardless of whether the athlete throws overhand or underhand.

For example, if PCA shows high variability in wrist deviation among underhand throwers, targeted neuromuscular or proprioceptive training may be prescribed to reduce compensatory errors. Similarly, consistent overhand patterns in shoulder mechanics can inform the design of assistive stabilizing equipment or classification benchmarks. Thus, PCA becomes a diagnostic tool for movement efficiency rather than a comparative judgment between strategies.

In addition, the Lifting algorithm consistently produced lower joint angle variability across throwing phases, improving the reliability of temporal and angular metrics used in performance monitoring. This enhanced stability supports more personalized coaching feedback and can serve as a foundation for real-time visual feedback systems—particularly valuable in settings where access to high-end motion capture is limited.

However, the underlying AI models were originally trained on able-bodied datasets, which may not fully capture the anatomical variability of athletes with disabilities. Although we fine-tuned ViTPose using images of seated boccia athletes (Methods Stage 3, Figure 3), some estimation errors may persist, particularly in joints or phases with atypical movement patterns. We recognize this as a limitation and highlight the need for future development of models trained on more representative adaptive sports datasets.

Beyond performance, this study contributes to fairness and objectivity in functional classification systems used in adaptive sports. Traditional classification relies heavily on human observation, which may overlook subtle but impactful differences in joint control. AI-supported biomechanical metrics, such as variability in elbow extension or shoulder trajectory, offer a transparent and data-driven supplement to subjective evaluations. This aligns with recent efforts to refine sport-specific classification with greater functional resolution [20].

The study also holds implications for rehabilitation and assistive technology development. AI-based motion analysis can support therapists in customizing treatment goals, tracking patient progress, and identifying compensatory movement patterns that may impact long-term joint health [21,22]. Moreover, the inclusion of paraprofessionals, such as trained aides or coaching assistants, in using such AI tools could help bridge implementation gaps in resource-limited settings—extending the benefits of technology-enhanced coaching to more diverse athletes.

Nonetheless, implementation challenges remain. Athletes often face logistical and financial barriers in accessing motion analysis technologies, especially within community or school-based programs. Spatial constraints and limited resources have been shown to hinder technology adoption and training opportunities in sports contexts [23]. To address this, future development should prioritize low-cost, portable AI tools and include paraprofessionals in capacity-building programs to support technology delivery at scale. In addition, the UNESCO report Impact Investment in Sport: Innovating the Funding of Sport for Development highlights the importance of public funding and cross-sector collaboration in ensuring that assistive sports technologies are accessible to all [24]. Broader adoption also requires open-source datasets featuring athletes with various disabilities. In addition, future studies should aim to include more balanced datasets that enable exploration of potential gender differences in throwing biomechanics. This is particularly important as the present study did not include validation of 3D pose reconstructions against a gold-standard system such as Vicon or Xsens, which represents a key limitation. Future work should aim to systematically assess the accuracy of AI-based pose estimation under seated adaptive sports conditions, using such validation to improve the generalizability and fairness of pose estimation models in diverse populations.

While this study does not propose new algorithms for pose estimation or biomechanical modeling, it addresses an important gap by applying and tailoring existing AI-based motion analysis tools to the specific needs of adaptive sports, particularly seated boccia. This applied and translational focus aims to support more accessible, practical solutions for coaching, athlete classification, and assistive technology evaluation within this underrepresented population.

This study is the first to unify OpenPose, ViTPose, and Lifting in a single framework for seated boccia analysis and to employ both DTW and PCA for strategy comparison and variability quantification. The five-phase segmentation offers a reference model for systematic training, and the joint-specific analysis opens new pathways for individualized coaching, equipment design, and classification validation.

In summary, this work bridges technological innovation and inclusive practice by offering a scalable, interpretable, and athlete-centered approach to performance evaluation. It paves the way for AI-assisted motion analysis to become a core component of personalized coaching, inclusive sport development, rehabilitation planning, and equitable classification in adaptive athletics.

## 5. Conclusions

This study developed a multi-stage, AI-powered pose estimation framework integrating OpenPose, ViTPose, and Lifting to analyze seated boccia biomechanics, improving accuracy in joint detection and 3D reconstruction. Results showed greater movement stability in overhand strategies and highlighted that biomechanical variability should be interpreted within individual functional profiles, not across throwing types. Beyond performance assessment, this approach holds strong potential in rehabilitation and assistive technology design by supporting therapists in setting goals, tracking progress, and identifying compensatory movements. Importantly, involving trained paraprofessionals in implementing AI tools could expand their accessibility and utility in resource-limited settings. Nonetheless, spatial constraints and resource shortages remain key challenges. To maximize impact, future work should focus on building representative seated-athlete datasets, enhancing real-time feedback systems, and developing portable, low-cost AI tools supported by paraprofessional training—advancing inclusive sports, personalized rehabilitation, and outcome-based evaluation of assistive technologies.

## Figures and Tables

**Table 1:** Standard deviation of joint angles in overhand and underhand throwing.

System	Joint	Overhand Athletes (°)	Underhand Athletes (°)
OpenPose	shoulder	6.8	7.2
	elbow	6.5	7.4
	wrist	5.9	6.8
Lifting	shoulder	3.5	3.9
	elbow	3.1	3.5
	wrist	2.7	3.2

## Data Availability

Data will be made available on request. The processed dataset contains identifiable segments and is subject to privacy considerations.
